# Dynamics and Differences in Systemic and Local Immune Responses After Vaccination With Inactivated and Live Commercial Vaccines and Subsequent Subclinical Infection With PRRS Virus

**DOI:** 10.3389/fimmu.2019.01689

**Published:** 2019-08-06

**Authors:** Miroslav Toman, Vladimir Celer, Lenka Kavanová, Lenka Levá, Jitka Frolichova, Petra Ondráčková, Hana Kudláčková, Kateřina Nechvátalová, Jiri Salat, Martin Faldyna

**Affiliations:** ^1^Department of Immunology, Veterinary Research Institute, Brno, Czechia; ^2^Faculty of Veterinary Medicine, University of Veterinary and Pharmaceutical Sciences, Brno, Czechia; ^3^Department of Virology, Veterinary Research Institute, Brno, Czechia

**Keywords:** porcine reproductive and respiratory syndrome, virus, antibody, cell-mediated immunity, inactive vaccine, modified-live vaccine

## Abstract

The goals of our study were to compare the immune response to different killed and modified live vaccines against PRRS virus and to monitor the antibody production and the cell mediated immunity both at the systemic and local level. In the experiment, we immunized four groups of piglets with two commercial inactivated (A1—Progressis, A2—Suivac) and two modified live vaccines (B3—Amervac, B4—Porcilis). Twenty-one days after the final vaccination, all piglets, including the control non-immunized group (C5), were i.n., infected with the Lelystad strain of PRRS virus. The serum antibody response (IgM and IgG) was the strongest in group A1 followed by two MLV (B3 and B4) groups. Locally, we demonstrated the highest level of IgG antibodies in bronchoalveolar lavages (BALF), and saliva in group A1, whereas low IgA antibody responses in BALF and feces were detected in all groups. We have found virus neutralization antibody at DPV 21 (days post vaccination) and higher levels in all groups including the control at DPI 21 (days post infection). Positive antigen specific cell-mediated response in lymphocyte transformation test (LTT) was observed in groups B3 and B4 at DPV 7 and in group B4 at DPV 21 and in all intervals after infection. The IFN-γ producing lymphocytes after antigen stimulation were found in CD4^−^CD8^+^ and CD4^+^CD8^+^ subsets of all immunized groups 7 days after infection. After infection, there were obvious differences in virus excretion. The virus was detected in all groups of piglets in serum, saliva, and occasionally in feces at DPI 3. Significantly lower virus load was found in groups A1 and B3 at DPI 21. Negative samples appeared at DPI 21 in B3 group in saliva. It can be concluded that antibodies after immunization and infection, and the virus after infection can be detected in all the compartments monitored. Immunization with inactivated vaccine A1—Progressis induces high levels of antibodies produced both systemically and locally. Immunization with MLV-vaccines (Amervac and Porcilis) produces sufficient antibody levels and also cell-mediated immunity. After infection virus secretion gradually decreases in group B3, indicating tendency to induce sterile immunity.

## Introduction

Porcine reproductive and respiratory syndrome (PRRS) is the most economically significant infectious disease currently affecting swine worldwide. Typical clinical symptoms of PRRS are mild to severe respiratory disease in infected newborn and growing pigs, and reproductive failure in pregnant sows. Two genotypes of the PRRS virus (PRRSV) have been identified: European (type 1) and North American (type 2). There are considerable genetic and virulence differences between and within PRRSV genotypes (1–3) correlated with a lack of cross-protection by vaccines (4–8). Highly pathogenic strains of PRRSV (HP-PRRSV) have been identified within both genotypes (9–11). Depending on viral strain and immune status of the host, some swine farms may have pigs subclinically infected, whereas others experience severe reproductive, and/or respiratory disease. Infection with both “classical” and highly pathogenic strains is associated with aberrant host immune response (9, 12).

Swine are the only known natural host of PRRSV and the primary target cells for replication of PRRSV are porcine alveolar macrophages (PAMs) (13). The first stage is represented by acute infection, resulting in viremia 6–12 h post-infection (PI), and lasting for several weeks despite the presence of circulating antibodies. In the second, persistent stage of infection, the virus is no longer detected in blood and lungs, and pigs no longer exhibit signs of clinical disease. In this stage, viral replication is primarily localized in lymphoid organs, including tonsils, and lymph nodes (14).

Infection with PRRSV elicit poor innate and adaptive immune responses associated with immune modulation and incomplete viral clearance in most of the pigs, depending on their age, and immune status (12, 15–17). Infection with certain PRRSV strains induced significant suppression of NK cell cytotoxic activity (18). The quantity of pro-inflammatory cytokines is significantly lower than in other viral infections and is strain dependent (19). PRRSV is also a poor inducer of IFN-α. Infection with PRRSV induces an antibody response (production) by 7–9 DPI but with no evidence of protection against PRRSV infection; serum neutralizing antibodies appear only later, typically ≥28 days PI (20). The virus also evades host cell-mediated immunity most likely by the promotion of immunosuppressive cytokines IL-10 and TGF-β resulting in delayed onset of Th1 immune response (18). Similarly, an immunosuppressive function of PRRSV was shown to probably be mediated by the cytokines IL-10 and TGF-β and action of Treg (21–23). Immunosuppression induced by PRRSV facilitates other viral and bacterial infections (18, 24, 25).

Vaccination is the principal means used to control and treat PRRSV infection. Several comprehensive review articles have been published recently. They critically evaluate different vaccination approaches against the PRRS virus and indicate the main weaknesses of current vaccines and vaccination strategies (26–29). Among others the problem are caused by high heterogeneity and occurrence of highly pathogenic strains and therefore efforts have been made to develop vaccines with a broad spectrum of effects (4, 5, 7, 30–33). However, the opinion still prevails that vaccination is more cost-beneficial over other health interventions (34–36).

Our study had the following three aims:

1. to establish complex immune response characteristics using several methodological approaches;
2. to monitor the dynamics in different compartments and in a time-dependent manner after vaccination and the challenging infection;
3. to compare the types of immune responses after vaccination with inactivated or live attenuated vaccines and subsequent challenge using a homologous strain.

## Materials and Methods

### Animals

Twenty-five weaned piglets aged 8 weeks and weighing 8–12 kg of the Large White breed from a PRRSV negative herd were used. The negative status of the animals was confirmed by serology using commercial ELISA kit (Idexx Labs). The use of animals was approved by the Branch Commission for Animal Welfare of Ministry of Agriculture of the Czech Republic (approval protocol No. MZe-1487) as a part of project as a part of project Respig (QJ1210120).

### Vaccines

Four commercial vaccines were used. Their characteristics are in Table 1.

**Table 1:** Characteristics of vaccines used in the experiment.

**Name**	**Producer**	**Type**	**Group of animals**	**Virus strain**	**Adjuvans**
Progressis	Merial	Inactivated	A1	P120	Water in oil
Suivac PRRS-In	Dyntec	Inactivated	A2	VD-E1, -E2, -A1	Water in oil saponin
Amervac PRRS	Hipra	Modified live	B3	VP-046BIS	Diluent A3 levamisole
Porcilis PRRS	Intervet	Modified live	B4	DV	Diluent Diluvac forte

### Challenge Virus

Lelystad strain PRRSV (CAPM V-490) was obtained from the collection of animal pathogenic microorganisms (CAPM) at the Veterinary Research Institute (Brno, Czech Republic). The virus was propagated on the MARC-145 cell line and maintained in Dulbecco's Modified Eagle's Medium (DMEM) (Invitrogen) supplemented with 10% fetal bovine serum (FBS) (Thermo Scientific), 1% antibiotics (Antibiotic Antimycotic Solution 100x: 10,000 units penicillin, 10 mg streptomycin, and 25 μg amphotericin B per mL; Sigma-Aldrich) at 37°C and 5% CO_2_. The virus was clarified by centrifugation, and its concentration was determined by plaque assay. The concentration of stock virus used in experiments was 5 × 10^6^ plaque forming units per mL.

### Experiment Design

Twenty-five piglets were used in the experiment. The piglets were assigned to five groups of five animals each according to weight and gender. The animals were housed in BSL2 isolation rooms, keeping animals from only one experimental group in each room. The animals were left to acclimate for 14 days after stocking. All piglets were clinically healthy at the time the experiment started. On day 0 (D0) two groups of piglets (A1 and A2) were immunized. Each animal was administered 2 ml of inactivated vaccine by an intramuscular (i.m.) injection. After 21 days (D21), piglets in these groups were revaccinated with the same dose, and piglets from the other two groups (B3 and B4) were immunized with 2 ml of a MLV vaccine. The health status of piglets was monitored on a regular basis, including temperature measurements, and samples of blood and other body fluids were taken for respective examinations at pre-set time intervals. After an additional 21 days (D42), all pigs, including control group (C5), were infected with 2 ml of the live PRRS virus. The piglets were monitored for another 21 days and then slaughtered (D63). Euthanasia was performed by exsanguination after combined anesthesia with a TKX (Telazol-Ketamin-Xylazin) mixture containing 12.5 mg/mL tiletamine and 12.5 mg/mL zolazepam (Telazol, Virbac, Carros, France), 12.5 mg/mL ketamine (Vetoquinol, Lure, France), and 12.5 mg/mL xylazine (Bioveta, Ivanovice na Hane, Czech Republic), administered intramuscularly in a final volume of 0.2 mL/kg body weight. As well as collection of blood and other body fluids (intestinal contents, bronchoalveolar lavage), an autopsy was performed and organs (lung parenchyma, spleen, lymph nodes,…) were collected for virological examination.

### Sampling

Blood samples for serum and heparin-treated blood samples were taken from the jugular vein. Group saline samples were collected using ropes which were left in the hutch for 3 h. Individual fecal samples were collected when handling the animals.

Bronchoalveolar lavage fluid (BALF) sampling was performed for the first time on live animals and for the second time after slaughter. The intravital lavage was performed with the animals under general anesthesia (a mixture of Xylazine and Ketamin) without the use of an endoscope by a method described earlier (37). Pigs were positioned in the sternal recumbency. An endotracheal tube was inserted into the trachea and 20 ml of sterile PBS (pH 7.2) was injected into the distal parts of the airways, toward the bronchus. About 60% of the infused saline was recovered as BALF aspirate and was filtered and centrifuged for 15 min at 200 g. Supernatant was stored at −20°C prior to serological analyses.

### Quantitative RT-PCR for Viral Load Detection

Total RNA from experimental samples of sera, oral fluids, and BAL (100 μL) was extracted using a NucleoSpin® RNA II kit (MACHEREY-NAGEL), in accordance with the manufacturer's instructions (protocol for total RNA preparation from biological fluids). The RNA obtained was eluted in 60 μL RNase-free water and immediately used for qRT-PCR amplification. Remaining RNA was frozen at −80°C for subsequent use.

Isolated RNA was used for qRT-PCR amplification by EZ-PRRSV™ MPX 4.0 Real Time RT-PCR kit (Tetracore), in accordance with the manufacturer's instructions. Quantification of the virus genome copies was based on quantification standards included in the kit.

### Serology Evaluation

For the evaluation of systemic and local antibody production two ELISA methods were used.

All swine sera tested were examined by commercially available ELISA test INGEZIM PRRS UNIVERSAL (Ingenasa), in accordance with the manufacturer's instructions, to examine for the presence of N protein specific IgG antibodies.

All swine sera, oral fluids, and BAL tested were examined by home-made indirect ELISA test based on recombinant nucleocapsid protein N of PRRS virus (developed previously in our department) for detection of specific IgM, IgG, and IgA antibodies.

Optimal antigen, serum, and antibodies concentrations were determined by checkerboard titration of positive and negative porcine sera. The cut-off value was determined by defining the upper prediction limit based on the upper tail of the t-distribution of negative control OD readings, at a confidence level of 99.5%. Positive serum with an absorbance corresponding to the calculated cut-off was included in all test plates.

The recombinant N protein diluted in 50 mM Bicarbonate-Carbonate Buffer pH 9.6 to a final concentration of 1.5 μg/mL was coated on 96-well-microtiter plates (Maxisorp II® Immunoplates, Nunc, Denmark) overnight at 26°C. The wells were then blocked with 3% skimmed milk in PBS for 90 min at 37°C and then washed with PBS. Positive and negative controls were included in each test plate. Each sample diluted in 3% skimmed milk in T-PBS (PBS with 0.1% Tween 20 and 0.5 M NaCl) was added in duplicates on antigen-coated wells with some differences among different types of samples. One hundred microliters of serum samples diluted 1:40 (for detection of IgM antibodies) or 100 μL of non-diluted samples of BAL (for detection of IgG and IgA antibodies) were incubated for 60 min at 37°C. Two hundred and fifty microliters of oral fluids diluted 1:2 were incubated 16 h at 4°C (for detection of IgG antibodies). Subsequently the plates were washed three times with T-PBS and antibody binding was detected by incubation for 60 min at 37°C with 100 μL of anti-Pig IgM peroxidase conjugate (1:10,000, Bethyl), anti-Pig IgG peroxidase conjugate (1:30,000, Sigma), or with anti-Pig IgA peroxidase conjugate (1:3,000, Bethyl) separately (diluted in T-PBS with 3% skimmed milk). After washing the plates as described above, 100 μL per well of the TMB-Complete (TEST-LINE) substrate was added. The optical density (OD) was measured at 450 nm after an incubation time of 5–10 min at room temperature.

### Virus Neutralization Test

The virus neutralization test for detection of PRRSV neutralization antibodies was performed as follows. Samples of sera were diluted 1:4 in DMEM medium (Sigma–Aldrich) supplemented with 3% FBS. Then, heat inactivated sera (56°C for 60 min) were diluted 2-fold serially in flat-bottom 96-well-microplate (NUNC). Next, equal volume (50 μL) of media containing 50 PRRSV PFU (Lelystad—CAPM V-490) was added to each well. Following incubation (60 min at 37°C) MARC-145 cells were added to each well (3 × 10^4^ per well) in 100 μL media per well. After 5 days of cultivation (37°C, 5% CO_2_), the cytopathic effect (CPE) of PRRSV on MARC-145 was evaluated by optical microscopy. The reciprocal value of the last sera dilution causing 50% reduction of CPE was defined as virus neutralization antibody titer.

### Lymphocyte Transformation Test

The lymphocyte transformation test was performed according to the method published earlier (38). Peripheral blood mononuclear cells (PBMC) were obtained by gradient centrifugation (Histopaque-1077, Sigma–Aldrich). Concentration of the cells was adjusted to 200,000 cells in 200 μL of RPMI-1640 medium supplemented with 10% of autologous serum, 100 IU/mL penicillin, 100 μg/mL streptomycin, and 4 μg/mL gentamicin). They were incubated with the 20 μg of optimal concentration of the specific antigen (MOI 1.0) for 5 days at 37°C in 5% CO_2_. Negative controls were incubated with RPMI-1640 medium only. All samples were evaluated in triplicate. ^3^H-thymidine was added on the last day of cultivation. Subsequently, the cells were harvested (FilterMate Harvestor, Packard Bioscience Company, USA), and ^3^H-thymidine incorporation was measured by a microplate scintillation and luminescence counter (TopCount NXT^TM^, Packard Bioscience Company) in counts per minute (CPM). The results were expressed in terms of stimulation indexes (SI), which were calculated as the ratio of CPM in stimulated samples vs. CPM in non-stimulated controls.

### ELISpot (for IFN-γ Production)

The number of IFN-γ producing cells was calculated by ELISpot techniques. Commercially available Porcine IFN-γ ELISpot kit (3130-4HPW-10, MABTECH) was used in accordance with the manufacturer's instructions. The number of cells used in the test was 5 × 10^5^/well. PRRSV was used for stimulation of the antigen-specific response in multiplicity of infection (MOI) 0.5. Mitogen ConA at a concentration 66 μg/mL was used as a positive control. Cells without stimulation were used as a negative control. Incubation lasted for 20 h. Spots were detected using ELISpot reader system ELRO7TL (AID, Germany). The results were recalculated to the number of CD3^+^ lymphocytes.

### Identification of Lymphocyte Subpopulation Producing IFN-γ After Antigen Stimulation

The 5 × 10^5^ of PBMC per well was stimulated with PRRS virus in MOI 0.5 for 20 h. The 5 × 10^5^ of cultured PBMC were pelleted and 20 μL of primary monoclonal antibody cocktail containing anti-CD4 (IgG2b, clone 74-12-4, WSU, Monoclonal Antibody Center, USA), anti-CD8 (IgG2a, clone 76-2-11, WSU, USA), and anti-γδ TCR (IgG1, clone PGBL22A, WSU, USA) and 20 μL of heat-inactivated goat serum was added. The cells were incubated for 20 min at 4°C and then rinsed twice with cell washing solution. Then, 50 μL of goat anti-mouse secondary antibody cocktail (anti-IgG2b: DyLight 405, anti-IgG2a: Alexa Fluor 647, and anti-IgG1: PE-Cy7) was added and the cells were incubated for another 20 min at 4°C. The cells were rinsed and then 70 μL of anti-CD3 antibody (IgG1, clone PPT3, Southern Biotech, pre-stained with Alexa Fluor 488 dye using Zenon Antibody Labeling Kit, Invitrogen) was added and the cells were incubated, rinsed twice, and fixation and permeabilization for subsequent intracellular staining was performed by solutions A and B of Intra Stain Kit (DAKO Cytomation, USA) (39). Finally, 5 μL of RPE-conjugated anti-IFN-γ antibody (clone CC302, AbD Serotec UK) was added and the cells were incubated for 30 min. The cells were measured as soon as possible using BD LSR Fortessa flow cytometer (Becton-Dickinson, USA). At least 100,000 events were acquired. The post-acquisition analysis of data was performed using the FACS Diva software (Becton-Dickinson, USA). The following lymphocyte subpopulations were identified: (CD3^+^) γδ^+^CD8^+^, (CD3^+^) γδ^+^CD8^−^, (CD3^+^γδ^−^) CD4^+^CD8^+^, (CD3^+^γδ^−^) CD4^−^CD8^+^, (CD3^+^γδ^−^) CD4^+^CD8^−^, (CD3^+^γδ^−^) CD4^−^CD8^−^, and CD3^−^CD8^+^. The percentage of IFN-γ-positive cells was established for each subpopulation.

### Statistical Analysis

The normality of data distribution were confirmed. Experimental groups were compared using non-parametric Man-Whitney test. Data from different dates were compared using non-parametric Wilcoxon test for paired samples.

### Legend on the Figure

Groups of five piglets were immunized i.m. with inactivated vaccine A1 (Progressis) or A2 (Suivac PRRS-In) at intervals D0 and D21. Groups of five piglets were immunized i.m. with modified live vaccine B3 (Amervac PRRS) or B4 (Porcilis PRRS) at interval D21. All animals were infected with a challenge virus on D42, including the group of control non-immunized piglets (C5).

## Results

### Detection of Antibody Levels in Sera

**Figure 1:**
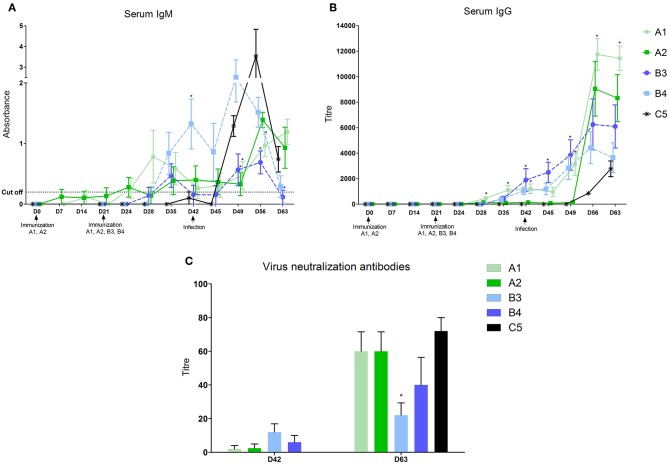
Levels of antibodies in sera. Levels of antibodies were measured in sera with home-made ELISA (**A**–IgM) or the commercial ELISA test Ingezim PRRS universal (**B**–IgG) Levels of virus neutralization antibodies 21 days after vaccination and 21 days after infection **(C)**. *statistically significant difference (*p* < 0.05) from control group.

After vaccination with inactivated vaccines (A1 and A2) the first IgM in the serum started to appear 14 days after the first dose in some piglets, and 7 days after the second dose in all animals of the A1 group (Figure 1A and Table 2). IgG antibodies appeared in all animals of both groups 7 days after the second dose (Figure 1B). The level of antibodies in the A1 group was significantly higher than in the group given the A2 vaccine. In groups of piglets vaccinated with MLV vaccines (B3 and B4), both IgM and IgG antibodies appeared 14 days after vaccination. On day 21 after immunization, their antibody responses were comparable to that of the A1 group.

**Table 2:** Summary of immune responses and virus excretion of individual vaccines used in the experiment.

	**Parameters**	**Time**	**A1—inactivated** **(Progressis)**			**A2—inactivated** **(Suivac)**			**B3—mlv** **(Amervac)**			**B4—mlv** **(Porcilis)**		
**ANTIBODY PRODUCTION**														
Serum	Dynamics: IgM	D24	+			+			–			–		
		D28	++			+			–			–		
		D35	++			+			++			+		
	Dynamics: IgG	D28	++			+			–			–		
		D35	++			+			++			++		
		D49	++			+			++			++		
		D63	+ + +			+ + +			++			++		
	Virus neutralization Ab	D42	±			±			++			+		
		D63	+ + +			+ + +			+			++		
Local response	Saliva IgG	D35	+			–			–			–		
		D49	++			–			+			–		
		D63	+ + +			++			++			++		
	BALF IgG	D35	+ + +			+			+			–		
		D63	++			++			+			+		
	BALF IgA	D35	++			++			++			+		
		D63	–			+			+			+		
**CELL MEDIATED IMMUNITY**														
	LTT: non-stimulated cells	All	+ + +			+ + +			++			–		
	LTT: stimulated cells (SI)	All	not evalable			not evaluable			+ at D28			++ at D28, D42, D49, D63		
	IFNγ in Ag–stimulated subsets	D49	positive CD4^−^CD8^+^cells			positive CD4^−^CD8^+^cells			positive CD4^−^CD8^+^cells			positive CD4^−^CD8^+^cells		
			positive CD4^+^CD8^+^cells			positive CD4^+^CD8^+^cells			positive CD4^+^CD8^+^cells			positive CD4^+^CD8^+^cells		
	Elispot IFNγ	D63	positive			positive			positive			positive		
**VIRUS LOAD**														
			serum	saliva	feces	serum	saliva	feces	serum	saliva	feces	serum	saliva	feces
	Post immunization	D24	–	–	–	–	–	–	++	++	–	++	++	–
		D42	–	–	–	–	–	–	+ + +	++	–	++	–	++
	Post infection	D45	++	++	+ + +	+ + +	–	+ + +	+ + +	++	+ + +	+ + +	++	+ + +
		D56	+	++	–	++	–	+	++	–	++	++	++	++
	The end of experiment	D63	+	++	++	+	++	+ + +	±	–	+	+	++	+

*         post vaccination (D0-D42) + + + positive (high); ++ positive (mid); + positive (low); ± positive in some animals*.

*         post infection (D45-D63) –negative*.

*   All    All intervals*.

*Groups of 5 piglets were immunized i.m with inactivated vaccine A1 (Progressis) or A2 (Suivac PRRS-In) at intervals D0 and D21. Groups of 5 piglets were immunized i.m with modified live vaccine B3 (Amervac PRRS) or B4 (Porcilis PRRS) at interval D21. All animals were infected with a challenge virus on D42, including the group of control non-immunized piglets (C5). All levels or activities after vaccination or infection are expressed as – (negative) or + to + + + as positive at different intensity*.

After infection, we identified a further increase in antibodies in the vaccinated groups. For the A1 group, a further increase in serum IgG antibodies was observed after 1 week and especially at 14 and 21 days after infection, when this antibody level significantly exceeded the values in the MLV immunized groups (B3 and B4) and the control one (C5). The A2 group showed a sharp increase on post infection days 14 and 21, and the level of serum IgG antibodies at these intervals was comparable to A1. In groups immunized with MLV (B3 and B4), serum IgG levels increased after 7, 14, and 21 days post infection, but did not reach the A1 group values. In the control, non-immunized group, the first IgM antibodies appeared 3 days after infection, with a significant increase on day 7 and 14. IgG antibody levels appeared 14 and 21 days after infection but were lower than in the immunized groups.

The virus neutralization antibody was detected in sera of animals 21 days after vaccination (Figure 1C). These antibodies were detected in some animals in the groups A1 and A2 only. At the end of experiment (D63, 21 days post-infection) the high level of virus neutralization antibodies were detected in all groups except B3 group, in which significantly lower level was found (Figure 1C).

### Detection of Antibody Levels in Other Compartments

**Figure 2:**
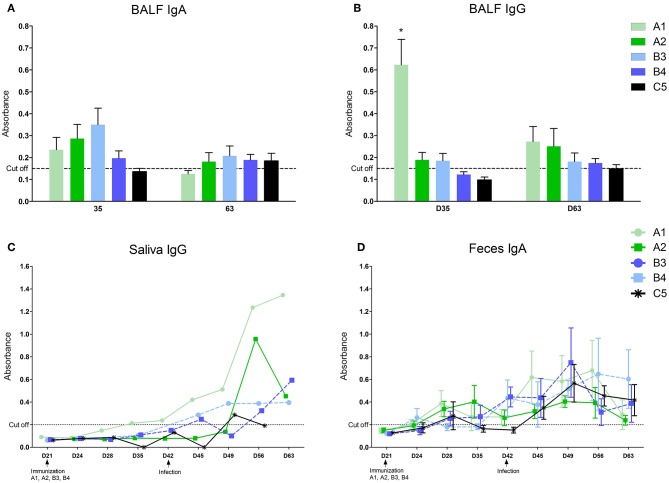
Levels of antibodies in different compartments. Levels of antibodies were measured in oral fluids **(A)**, fluid from bronchoalveolar lavages (**B**–IgG, **C**–IgA), and feces (**D**–IgA) with home-made ELISA. *statistically significant difference (*p* < 0.05) from control group.

Local antibodies in the BALF performed 14 days post vaccination we detected low levels of IgA in all immunized groups (Figure 2A) and IgG in A1, A2, and B3 (none in B4) with the level in the A1 group being significantly higher (Figure 2B). 21 days after infection we detected low levels of IgA antibodies in A2, B3, B4, and control group (C5) and low levels of IgG antibodies in all immunized groups (none in C5). Local antibodies were detected in the saliva of the A1 group in low concentrations 14 and 21 days after the second vaccination and in all groups after infection with variability in individual intervals and with no statistically significant between-group difference (Figure 2C). In the feces, local antibodies (IgA) were detected from 1 week after the second immunization dose in groups A1 and A2, and from 1 week after MLV immunization in groups B3 and B4 (Figure 2D) Increased levels of antibody were detected in all groups including control after infection with no statistically significant between-group difference.

### Cell Mediated Immune Response

**Figure 3:**
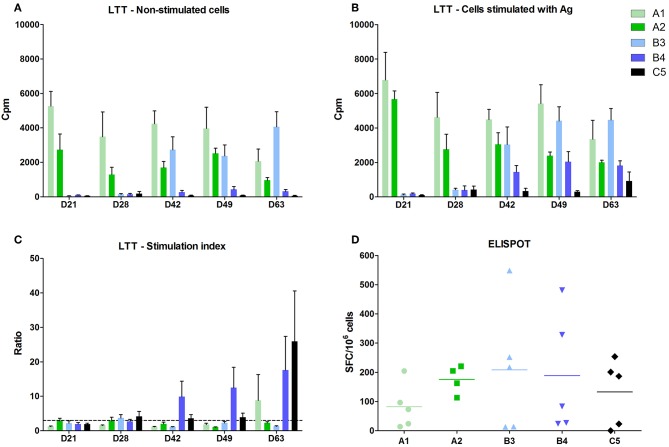
Cell mediated immunity. The activity of blood lymphocytes was measured in the lymphoblast transformation test (LTT) using 5-day cultivation of cells. **(A)** Control, activity of non-stimulated cells. **(B)** Activity of cells stimulated with PRRSV antigen. The activities were measured after adding ^3^H-thymidine and counted as counts per minute (CPM) in a beta-counter. **(C)** Ratio the ratio of stimulated to non-stimulated cells (stimulating index—SI). **(D)** ELISPOT. The number of IFN-γ producing cells was calculated in cells from bronchoalveolar lavage fluid (BALF) on D63, i.e. 21 days after the challenge infection. The results were recalculated to the number of CD3^+^ lymphocytes.

A positive cell-mediated response after lymphocyte stimulation with specific antigen *in vitro* (stimulation index in LTT above 3) was observed in the B4 group after 7 and 21 days post vaccination and 7 and 21 days post-infection (Figure 3). A positive stimulation index was detected in the B3 group at 7 days post vaccination only as a non-specific basal stimulation occurred from 21 days post vaccination in this group (Figure 3A).

Groups immunized with inactivated vaccines A1 and A2 showed a marked non-specific stimulation of cells even without using antigen (Figure 3A) and, therefore, it was impossible to demonstrate the effect of antigen addition and thus cell-mediated immune response.

Cell-mediated immune response after challenge infection was positive in all vaccinated groups and in the control group after 21 days post infection, using ELISpot in PBMC from bronchoalveolar lavages. The results were recalculated to the number of CD3^+^ lymphocytes. The differences between individual animals, but no significant differences between groups were detected (Figure 3D).

We detected IFN-γ producing lymphocytes after PRRS antigen stimulation. The most marked differences from control were found in CD4^−^CD8^+^ and CD4^+^CD8^+^ (and partly also in CD3^−^8^+^ and γδ^+^8^+^) subsets of all immunized groups 7 days after infection.

### Virus Load and Clinical Signs

In the groups vaccinated with live vaccines (B3 and B4), the virus load was demonstrated in serum and saliva from day 3 after immunization, in BALF 14 days after immunization (the only time point when the lavage was taken), and in feces occasionally 7 days after vaccination, then in all piglets 14 days after immunization (data not shown).

No clinical signs were observed in piglets after infection. Elevated body temperature was occasionally found in the first 2 days, independent of the experimental group.

**Figure 4:**
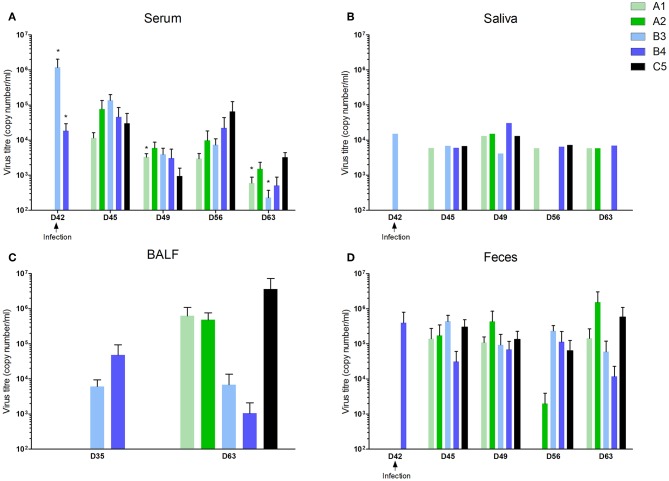
Viral load in different compartments. Groups of 5 piglets were immunized i.m with inactivated vaccine A1 (Progressis) or A2 (Suivac PRRS-In) at intervals D0 and D21. Groups of 5 piglets were immunized i.m with modified live vaccine B3 (Amervac PRRS) or B4 (Porcilis PRRS) at interval D21. All animals were infected with a challenge virus on D42, including the group of control non-immunized piglets (C5). Viral load was measured by quantitative real-time-PCR. **(A)** Viral load in sera, **(B)** oral fluids, **(C)** fluid from bronchoalveolar lavages, and **(D)** feces. *statistically significant difference (*p* < 0.05) from control group.

However, viral shedding was noted, with between-group differences. The virus appeared in serum, saliva, and feces in all groups including the control group 3 days after infection (Figures 4A,B,D). The virus was detected in BALF 21 days after infection in A1, A2, and C5 groups (Figure 4C). Virus shedding was decreased in immunized groups 14 and 21 days after infection with the level in the A1 and B3 group being significantly lower compared to control group 21 days after infection. Negative samples appeared 21 days after infection in saliva (in B3 group) and in feces (B3 and B4 groups).

## Discussion

The goals of our study were (1) to establish comprehensive immune response characteristics using several methodological approaches and monitor the dynamics in different compartments and in a time-dependent manner after vaccination and the challenging infection and (2) to compare the immune response to different killed and modified live vaccines against PRRS using these methodological tools. In order to compare the immune response after vaccination with different vaccines, we used a model of vaccinations of young piglets (beginning at 8 weeks of age) and given vaccination intervals and subsequent infections, regardless of the fact that manufacturers' recommendations were different (especially in Progressis).

There are only a few papers published providing a comprehensive picture of immune response after vaccination against PRRSV (40–43) because the majority of the existing studies are based mainly on the evaluation of the vaccination effectiveness by monitoring the immune responses found in the blood (5, 30, 33, 44–46). Our results show that antibodies after immunization and infection, and the virus after infection, can be detected in all the monitored compartments (blood, respiratory tract, intestine). By repeated sampling and simultaneous monitoring of the antibody and cell-mediated immunity and virus shedding systematically and locally, we have managed to get comprehensive information about the dynamics of the immune response after vaccination or PRRS virus infection.

In practical diagnostics of field samples is an effort to seek simple approaches to obtain tentative information on the epidemiological situation of the herd. One current trend is the monitoring of antibody levels and shedding of the virus in the oral fluid (41–43). In our experiment, the antibody detection rate in the oral fluid collected with ropes in pens was sufficient. The levels of antibodies detected after vaccination were low, but they increased after challenge infection. These findings confirms the possibility of using this approach for preliminary characteristics in the herd. It was interesting to observe the dynamics of antibody levels and viral shedding in feces too. This is an approach which is not often used for PRRSV infection monitoring but is used in other situations where feces samples are more readily available than samples from other sources (47, 48). We were wondering, among other things, to what extent infections occurring systemically, or locally in the respiratory tract occur in this remote compartment. Our findings show that fecal samples can also be used for PRRSV infection monitoring. Detection of both the viruses and antibodies is not entirely consistent, because they appear in individual animals, and cease at later intervals, therefore, it is necessary to consider these findings as approximate. They can be used for herd- or pen- level testing, but not for establishing a diagnosis in individual animals.

It appeared to be technically difficult to demonstrate specific cell-mediated immunity. The partial results were provided by each of the three methods used and a comprehensive picture could be obtained by compiling this information. Therefore, it was not possible to use only data of IFN-γ production in ELISpot, although it is currently the most commonly used method for CMI (5, 29, 37, 38, 49, 50). A positive cell-mediated response after lymphocyte stimulation with specific antigen *in vitro* (in lymphocyte transformation test) was observed in MLV groups and especially in the B4 group as a non-specific basal stimulation occurred from 21 days post-vaccination in B3 group. The strong non-specific stimulation of PBMC without specific antigen were detected in groups A1 and A2 immunized with inactive types of vaccine. This non-specific stimulation of cells *in vivo* masks the overall picture, and thus specific cell-mediated immunity cannot be demonstrated. This effect is attributed to the use of strong adjuvants in inactivated types of vaccines. In the test of IFN-γ production and detection with ELISpot, which is very often used to identify CMI both in experimental studies (5, 37), and in the field (49, 50), we have shown an increase in both blood and cells acquired by lavage, but the individual variability among the animals was too high and, consequently, there were no differences found between the groups under study. We detected also IFN-γ producing lymphocytes after PRRS antigen stimulation in all immunized groups 7 days after infection. The most marked differences from control were found in CD4^−^CD8^+^ and CD4^+^CD8^+^ (and partly also in CD3^−^CD8^+^ and γδ^+^CD8^+^) subsets of lymphocytes. The CD4^−^CD8^+^ subpopulation belongs to cytotoxic groups of cells, CD4^+^CD8^+^ is considered a group of Th1 memory cells (51). In another study the expression of cytotoxic CD4^+^CD8^+^ and CD4^+^CD8^−^ was described which help to recover from PRRS infection (52).

There were qualitative and quantitative differences in the immune responses to the inactivated vaccines and to MLV ones. After immunization with the inactivated vaccine (especially A1—Progressis), high levels of antibodies were produced generally (serum), which were mostly of the IgM, and IgG isotypes, and also locally (saliva, BALF), both IgG and IgA. Nevertheless it should be noted that we have applied Progressis to piglets in our study, while the manufacturers declare the use of this vaccine for gilts and sows. Cell-mediated immunity was detected only after infection, high non-specific cell stimulation was detected after vaccination and therefore any specific response could not be demonstrated in these intervals. The antigen specific cell mediated immunity after inactivated vaccine is rarely described (50). Most work describes low or no CMI after vaccination with inactivated vaccine.

After immunization with MLV vaccines, sufficient levels of antibodies in serum and BALF (IgG) were also produced, but lower than after the inactivated vaccine administration. The levels of IgA antibodies in BALF were comparable but low. Low levels of virus neutralization antibodies after vaccination can be explained by a short interval between vaccination and infection, since neutralizing antibodies after vaccination or PRRS infection occur within 28 days (42).

The dynamics of virus shedding after vaccination and infection is often used for monitoring vaccine efficacy (30, 40, 49). The decrease in virus secretion was observed 14 days after MLV immunization and disappearance in 28 days (42). In another study the excretion of virus was described still for 21 days after vaccination with for Porcilis or Amervac vaccine (53). Demonstration of cell-mediated immunity and reduction in viral load correlate with studies by other authors and support the preferred use of MLV vaccines in the control of PRRS infection (29, 46).

The question is to what extent these results are influenced by the composition of vaccines from different manufacturers and to what extent different types of vaccines (inactivated vs. live attenuated). There was an obvious difference in the quality between the inactivated vaccines, whereas the character of the immune response to both MLV vaccines was similar with only partial differences in the time-related response dynamics. The vaccine B3 (Amervac) showed a more pronounced decrease in virus secretion and a tendency to induce sterile immunity, while B4 (Porcilis) vaccine had a more pronounced of CMI in lymphocyte transformation test. It should be noted that the strain used in Porcilis had a higher genetic link with the Lelystad strain compared to the strain of Amervac (54, 55) which, however, probably did not significantly affect the above characteristics.

Despite the fact that many studies focused on PRRS immunoprophylaxis have already been published and many procedures are implemented in the agricultural industry, a universal model does not yet exist (46–60). The use of live attenuated vaccines is generally preferred as was also confirmed in our field study (61). In this study, we controlled the infection by a repeated blanket immunization with MLV vaccine (Porcilis), followed by targeted immunization of gilts, and sows. The success of the strategy selected and evidence of virus eradication from the given herd were demonstrated by introducing sentinel animals into a fattening herd. Based on this result, we believe that control programs can be adopted even in herds with continual throughput housing without interrupting production. However, in this case, vaccination is only one of the necessary preconditions and the introduction of very strict principles of good biosecurity is of no less importance.

## Ethics Statement

The use of animals was approved by the Branch Commission for Animal Welfare of Ministry of Agriculture of the Czech Republic (approval protocol No. MZe 1487) as a part of project as a part of project Respig (QJ1210120).

## Author Contributions

MT, VC, JS, and MF designed the study. MT, LL, JS, and KN performed the experiments. LL, HK, LK, PO, and JF performed the lab work and analyzed the data. LK produced the figures and statistical analysis. MT wrote the manuscript. JS and MF participated in manuscript preparation. All authors read and approved the final manuscript.

### Conflict of Interest Statement

The authors declare that the research was conducted in the absence of any commercial or financial relationships that could be construed as a potential conflict of interest.
